# P-137. Contención de la resistencia a los antimicrobianos en entornos de recursos limitados: la experiencia de Argentina en la Red de Acción Global en Salud - Módulo de Resistencia a los Antimicrobianos

**DOI:** 10.1093/ofid/ofae631.342

**Published:** 2025-01-29

**Authors:** Angel M Colque, Laura Alonso, Laura Barcelona, Irene Pagano, Paula Iantorno, Rachel Mann Smith, Maria Ines Staneloni, Veronica Berdiñas, Cecilia Echave, Andrea Gonzalez, Claudia Velasquez Carretero, Nora Fueyo, Fabiana Ponce, Tomas Fueyo, Alejandra Corso, Fernando Pasteran

**Affiliations:** Complejo Medico Churruca Visca, Ciudad Autonoma de Buenos Aires, Ciudad Autonoma de Buenos Aires, Argentina; Instituto Nacional de Epidemiologia “Dr Juan H. Jara” Anlis Malbran, Buenos Aires, Ciudad Autonoma de Buenos Aires, Argentina; Comisión Nacional de Resistencia a los Antimicrobianos, Buenos Aires, Ciudad Autonoma de Buenos Aires, Argentina; Instituto Nacional de Epidemiologia “Dr Juan H. Jara” Anlis Malbran, Buenos Aires, Ciudad Autonoma de Buenos Aires, Argentina; Comisión Nacional de Resistencia a los Antimicrobianos, Buenos Aires, Ciudad Autonoma de Buenos Aires, Argentina; CDC, Atlanta, Georgia; Center for Diseases Control and Prevention, Buenos Aires, Ciudad Autonoma de Buenos Aires, Argentina; Hospital Municipal Dr. Bernardo Houssay, Vicente Lopez, Buenos Aires, Argentina; Hospital General de Niños Pedro de Elizalde, Buenos Aires, Ciudad Autonoma de Buenos Aires, Argentina; Hospital Municipal Dr. Bernardo Houssay, Vicente Lopez, Buenos Aires, Argentina; Hospital Municipal Dr. Bernardo Houssay, Vicente Lopez, Buenos Aires, Argentina; Hospital General de Niños Pedro de Elizalde, Buenos Aires, Ciudad Autonoma de Buenos Aires, Argentina; Hospital General de Niños Pedro de Elizalde, Buenos Aires, Ciudad Autonoma de Buenos Aires, Argentina; Hospital General de Niños Pedro de Elizalde, Buenos Aires, Ciudad Autonoma de Buenos Aires, Argentina; ANLIS-Malbrán, Buenos Aires, Ciudad Autonoma de Buenos Aires, Argentina; ANLIS-Malbrán, Buenos Aires, Ciudad Autonoma de Buenos Aires, Argentina

## Abstract

**Background:**

The Global Action in Healthcare Network, antimicrobial resistance module (GAIHN-AR) is a CDC-led global network that partners with PAHO and Ministries of Health (MOH) in Argentina and Chile. Its goals are to prevent, detect, and respond to healthcare-related AR threats; it is a part of the CDC Global AR Laboratory and Response Network. We describe the containment response experience in 2 Argentine public hospitals participating in GAIHN-AR.

**Methods:**

GAIHN-AR containment guidance was adapted to the local context and targeted carbapenemase-producing Enterobacterales (CP-CRE) carrying novel (Tier 1) or known, but rare, carbapenemases (Tier 2: non-OXA-163 OXA-48 variants, VIM, multiple carbapenemases, and pan-drug-resistant [PDR]). Hospital laboratories implemented new diagnostics for carbapenemase identification and developed protocols to communicate alerts of potential Tier 1 or 2 CP-CRE to hospital infection prevention and control (IPC) teams, MOH, and the National Reference Lab (NRL). Hospitals received training and support from MOH GAIHN-AR staff to conduct containment and IPC activities. Containment activities continued while alert organisms were confirmed or discarded by the NRL.

**Results:**

During Jan 2023 -- March 2024, 20 alerts were generated (all Tier 2); 19 were not confirmed (17 were OXA-163, 1 was PDR non-confirmation, 1 was mixed culture). The remaining confirmed alert (NDM+KPC) generated a successful containment response with 7/7 contacts screening negative. With each alert, gaps were identified, particularly in communication across stakeholders and hospital IPC activities. In response, enhanced monitoring was implemented for hand hygiene, contact precautions and environmental hygiene, and the MOH developed a new informatic system to facilitate stakeholder communication.

**Conclusion:**

Combating AR threats requires support and integration of IPC programs and clinical laboratories to ensure rapid communication and action when target organisms are detected. Support from CDC and PAHO was critical for implementation and MOH involvement is key for successful responses to AR threats. Although vital learning experiences, steps to limit false alerts are key to prevent response fatigue moving forward.

**Disclosures:**

**All Authors**: No reported disclosures

